# Electroless Deposition of Noble Metals on Rod-Shape Plant Viruses in Various Aqueous Metal Precursor Solutions

**DOI:** 10.1021/acsomega.4c01391

**Published:** 2024-08-08

**Authors:** Vindula Basnayake Pussepitiyalage, Che-yu Chou, Michael T. Harris, L. Sue Loesch-Fries, Shohreh Hemmati

**Affiliations:** † School of Chemical Engineering, Oklahoma State University, Stillwater, Oklahoma 74078, United States; ‡ Davidson School of Chemical Engineering, Purdue University, West Lafayette, Indiana 47907, United States; § Botany and Plant Pathology, Purdue University, West Lafayette, Indiana 47907, United States; ∥ School of Mathematics and Natural Sciences, The University of Southern Mississippi, Hattiesburg, Mississippi 39406, United States

## Abstract

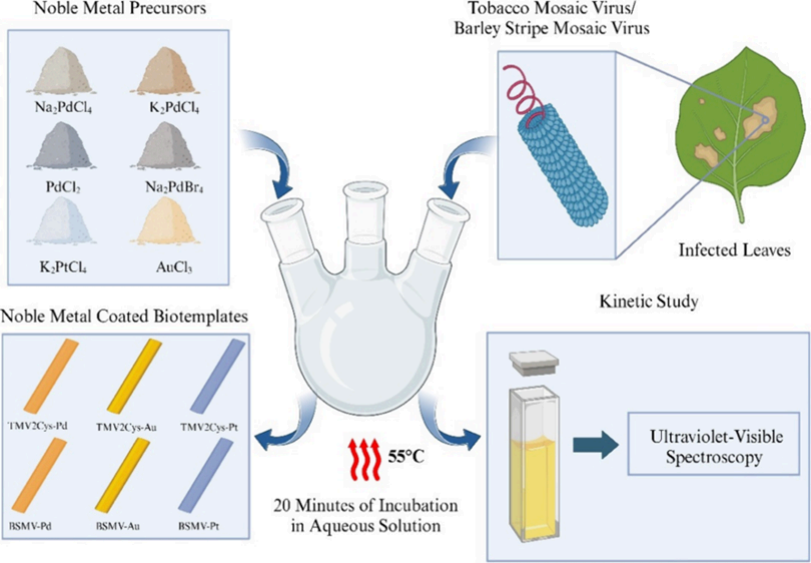

The challenge of synthesizing noble metal nanostructures sustainably has encouraged researchers to explore biological routes for nanostructure production, such as biotemplating. Plant viruses with rod-shape morphology, such as tobacco mosaic virus (TMV) and barley stripe mosaic virus (BSMV), offer promising biotemplates to produce metal nanorods. TMV and BSMV can be incubated in aqueous metal precursor solutions to mineralize metals on the coat proteins (CPs) of the viruses. Previous studies have primarily examined palladium (Pd) mineralization on TMV and BSMV using Na_2_PdCl_4_ as the Pd precursor. There is limited scientific literature on the effect of using alternative Pd precursor solutions besides Na_2_PdCl_4_ such as K_2_PdCl_4_ and PdCl_2_ to mineralize Pd on TMV and BSMV. Past attempts at mineralizing other noble metals such as platinum (Pt) and gold (Au) required an initial layer of Pd to be deposited on the TMV and BSMV biotemplates. In this study, we aimed to expand the understanding of using alternative Pd precursor solutions to mineralize Pd on TMV and BSMV. Additionally, the deposition of Pt and Au onto TMV and BSMV without the need for an initial Pd mineralization layer was achieved using alternative Pt and Au precursors, including K_2_PtCl_4_ and AuCl_3_, respectively. Pd, Pt, and Au were successfully deposited on TMV and BSMV by incubation in aqueous solutions of Na_2_PdCl_4_, K_2_PdCl_4_, PdCl_2_, K_2_PtCl_4_, and AuCl_3_. Kinetic studies were also conducted using ultraviolet–visible (UV–vis) spectroscopy to examine the rates at which Pd, Pt, and Au precursor ions were reduced during the mineralization process, mimicking their adsorption onto TMV and BSMV CPs. BSMV adsorbed noble metal precursor ions faster than TMV as determined by UV–vis spectroscopy. While palladium nanorods (PdNRs) offer high electrical conductivity desirable for electronic applications, Pd-coated TMV and BSMV may face limitations due to their organic cores, potentially compromising conductivity. To address this, one approach is to convert the organic core into conductive amorphous carbon through thermal annealing. In this study, *in situ* transmission electron microscopy was utilized to thermally anneal Pd-TMV2Cys, thereby transforming them into PdNRs with amorphous carbon cores.

## 1 Introduction

Noble metals such as palladium (Pd), platinum (Pt), and gold (Au) have a wide range of applications in science and technology due to their antimicrobial,^1^ conductive,^2^ corrosion resistive,^3^ and catalytic properties.^4^ Nanoscale utilization of noble metals has the potential to create applications in fields like biotechnology,^5^ food processing,^6^ and semiconductors.^7^ Nanoscale applications of noble metals require the production of Pd, Pt, and Au nanostructures with morphologies such as nanospheres,^8^ nanotubes,^9^ nanowires (NWs),^10^ and nanorods (NRs).^11^ The bottom-up chemical synthesis of 1-dimensional (1D) noble metal nanostructures, such as NWs and NRs, has captured the attention of researchers due to their application in electronics^12,13^_,_ catalysts,^14^ sensors,^15^ and drug delivery.^8^ 1D metal nanostructures are conventionally synthesized by using the polyol process. However, this process is unsustainable, because it uses toxic polyols such as ethylene glycol (EG) as the reducing agent and solvent to synthesize metal nanostructures.^16,17^ Biotemplating is a more sustainable approach to synthesizing 1D noble metal nanostructures because it foregoes the use of hazardous reagents and requires less energy. Biotemplates, such as viruses,^18,19^ virus-like particles (VLPs),^20^ bacteria,^21^ DNA,^22^ and other biological agents, have been used for the synthesis of metal nanostructures with noteworthy properties. Rod-shape viruses are of particular interest due to their application in the production of metal NRs.^18,23^ Rod-shape virions such as tobacco mosaic virus (TMV)^23^ and barley stripe mosaic virus (BSMV)^18^ have been shown to possess the surface properties for mineralization of metals on their capsid proteins (CPs). The amino acid residues present on the CPs, such as aspartic (Asp), cysteine (Cys), threonine (Thr), and glutamic (Glu) acid, contain functional groups like hydroxyl, carboxyl, and amine groups that undergo oxidation while reducing complex metal ions to form metal atoms.^18^ The formed metal atoms are deposited on the CPs surface; thereby resulting in metal mineralized biotemplates.^18,23^ TMV and BSMV can be extracted sustainably from natural sources; therefore, using these biotemplates is an environmentally sensitive procedure for metal NR synthesis. Metal mineralization on the surfaces of TMV and BSMV is possible without the use of an external reducing agent, in an aqueous solution, and at low temperatures. Therefore, TMV and BSMV can be used for the sustainable production of noble metal NRs, such as palladium nanorods (PdNRs),^18^ platinum nanorods (PtNRs),^24^ and gold nanorods (AuNRs).^25^ TMV and BSMV with metal mineralization on their CPs have been successfully employed in a range of applications, including but not limited to electronics,^3,26,18^catalysts,^27^ sensors,^28^ and energy storage.^29^

TMV is a rod-shape virus with a length of 300 nm and a diameter of 18 nm. The 2,130 CP subunits are 17.5 kDa and arranged helically with 49 subunits in three turns of the helix.^30^ The ribonucleic acid (RNA) of TMV is embedded around the central channel of the particle. TMV2Cys contains two additional cysteine amino acid residues added to positions 2 and 3 of the CP sequence as surface modifications. The additional cysteine residues facilitate more dense metal mineralization on the CPs of TMV2Cys.^31^ BSMV rod-shape particles are 110–150 nm in length and 20 nm in diameter. The 25 kDa CP subunits are helically arranged with 26 CP subunits per turn.^32^ TMV2Cys was explored due to improved metal mineralization promoted by the additional amino acid residues. BSMV without any additional amino acid residue modifications was also explored because BSMV adsorbs reacting metal ions using both covalent and electrostatic interactions. TMV relies solely on covalent interactions for metal ion adsorption; therefore, BSMV is a more promising biotemplate for metal mineralization than TMV.^18^

Previous studies have comprehensively examined the effect of Pd mineralization on TMV and BSMV using kinetic and parametric studies.^18,33,34^ Deposition of other metals such as cobalt (Co), silver (Ag), copper (Cu), and iron (Fe) on TMV and BSMV CPs has been challenging because Co^2+^, Ag^+^, Cu^+^, and Fe^2+^ ions have less positive reduction potentials compared to Pd^2+^. Therefore, Co, Ag, Cu, and Fe do not readily mineralize on TMV and BSMV similar to Pd.^33^ Previous studies have achieved a higher degree of success in mineralizing Au, Co, Fe, and Ni onto TMV by employing an initial Pd coating layer. This increased effectiveness can be attributed to the catalytic reduction of metal ions facilitated by the presence of Pd on the CPs.^3,25,35^ This study fine-tuned the mineralization of Au and Pt to deposit Au and Pt directly onto the TMV and BSMV without an initial Pd coating. In prior studies, the mineralization of Pd onto TMV and BSMV was achieved through the incubation of the particles in solutions containing Na_2_PdCl_4_.^18,20,36^ This study investigated the impact of employing metal precursor solutions other than Na_2_PdCl_4_ for depositing Pd onto TMV and BSMV. Transmission electron microscopy (TEM) characterization was employed to observe the mineralization of Pd, Pt, and Au on TMV and BSMV. Ultraviolet–visible (UV–vis) spectroscopy was used as an indirect measure of the progress of Pd mineralization to investigate the kinetics of adsorption and mineralization of Pd, Pt, and Au ion species onto TMV and BSMV. Our study introduces the use of alternative Pd precursors for the electroless deposition of Pd nanoparticles on TMV and BSMV, demonstrating several advantages over traditional precursors like Na_2_PdCl4 or PdCl_2_. These alternative precursors offer cost efficiency as well as higher deposition rates. Furthermore, we achieved the direct deposition of Pt and Au onto TMV and BSMV without the need for an initial Pd coating, simplifying the process and reducing the costs. Our investigation into the kinetics of metal nanoparticle mineralization provides novel insights, enhancing the versatility and applicability of TMV and BSMV as biotemplates.

## 2 Results and Discussion

### 2.1 TMV2Cys and BSMV Biotemplates

**Figure 1:**
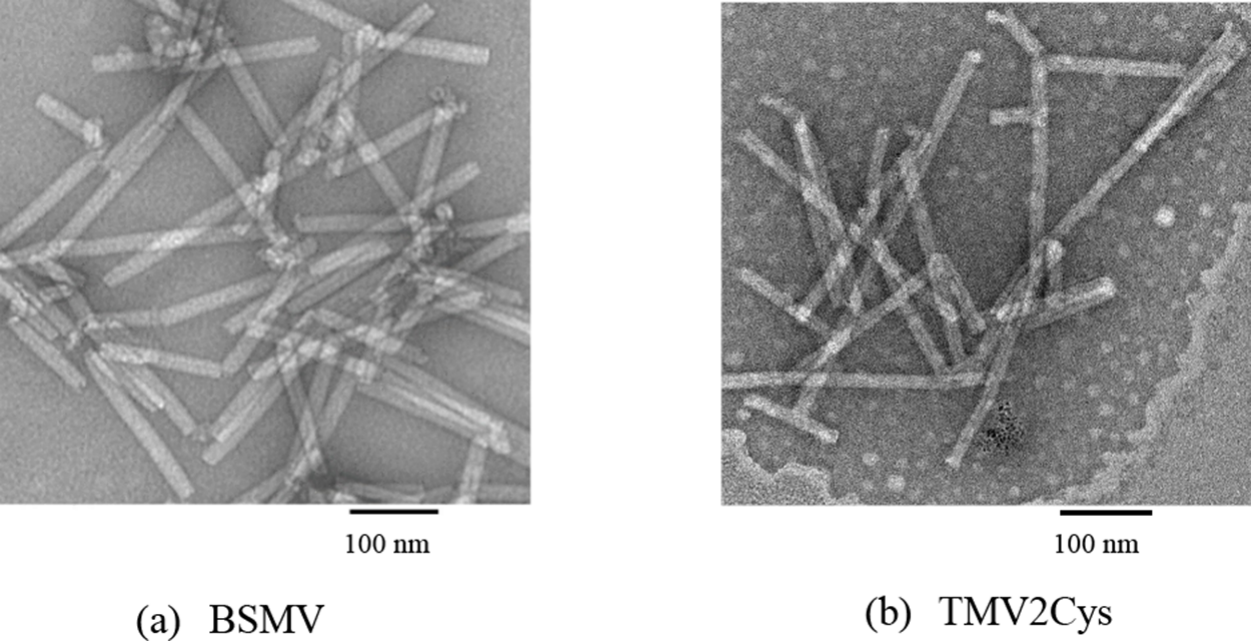
TEM images of BSMV (a) and TMV2Cys (b) without a metal coating.

TMV2Cys and BSMV were characterized using TEM as shown in Figure 1. The widths of the biotemplates were measured and recorded to be 20 ± 0.9 and 18 ± 0.7 nm for BSMV and TMV2Cys, respectively.

### 2.2 Palladium Mineralization on TMV2Cys and BSMV Using Different Pd Precursors

**Figure 2:**
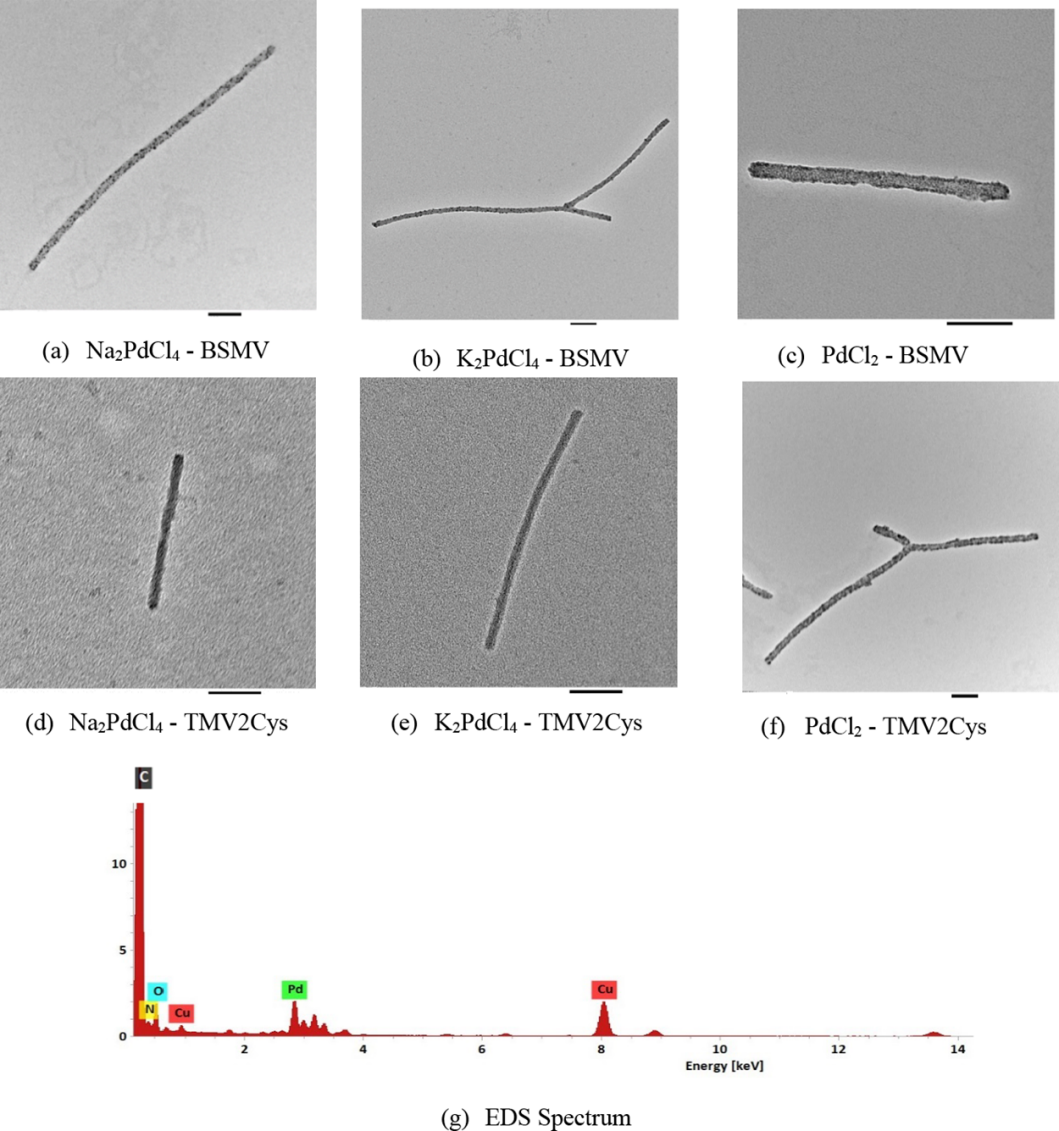
TEM images of Pd coated on BSMV produced by incubating BSMV in Na_2_PdCl_4_ (a), K_2_PdCl_4_ (b), PdCl_2_ (c), and Pd coated on TMV2Cys produced by incubating TMV2Cys in Na_2_PdCl_4_ (d), K_2_PdCl_4_ (e), and PdCl_2_ (f). EDS spectrum of pd-coated TMV2Cys in Na_2_PdCl_4_ ((g) scale bars: 100 nm).

TMV2Cys and BSMV were coated with Pd using the directions presented in the experimental details section. The images obtained from TEM characterization of Pd-coated BSMV (BSMV-Pd) and Pd-coated TMV2Cys (TMV2Cys-Pd) are displayed in Figure 2a–f. The presence of Pd mineralization on the surface of TMV2Cys incubated in Na_2_PdCl_4_ solution was further confirmed using energy-dispersive X-ray spectroscopy (EDS) characterization as shown in Figure 2g.

No Pd mineralization was observed when TMV2Cys and BSMV were incubated in Na_2_PdBr_4_ solution; therefore, the samples were not characterized using TEM. The average diameter of the BSMV-Pd and TMV2Cys-Pd produced using the different Pd precursor solutions were measured and are tabulated in Table 1. Based on Table 1, BSMV-Pd particles were thicker than TMV2Cys-Pd produced using each Pd precursor solution. The thickest layers of Pd deposition were observed on the CPs of BSMV-Pd and TMV2Cys-Pd that were produced by using PdCl_2_ as the metal precursor.

**Table 1:** Dimensions of Biotemplates with Metal Coating: Assessing the Diameter and Metal Thickness

biotemplate-metal	average diameter of biotemplate without metal mineralization	metal precursor	average diameter of biotemplate with metal mineralization	average thickness of metal mineralization
BSMV-Pd	20 ± 1.0 nm	Na_2_PdCl_4_	30.5 ± 1.2 nm	5.2 ± 1.6 nm
		K_2_PdCl_4_	30.2 ± 1.8 nm	5.1 ± 2.1 nm
		PdCl_2_	32.5 ± 2.0 nm	6.3 ± 2.2 nm
BSMV-Pt		K_2_PtCl_4_	34.1 ± 1.2 nm	7.0 ± 1.6 nm
BSMV-Au		AuCl_3_	34.7 ± 1.5 nm	7.4 ± 1.8 nm
TMV2Cys-Pd	18 ± 1.0 nm	Na_2_PdCl_4_	26.3 ± 1.9 nm	4.2 ± 2.1 nm
		K_2_PdCl_4_	25.9 ± 2.1 nm	4.0 ± 2.3 nm
		PdCl_2_	27.4 ± 2.4 nm	4.7 ± 2.6 nm
TMV2Cys-Pt		K_2_PtCl_4_	30.2 ± 1.7 nm	6.1 ± 2.0 nm
TMV2Cys-Au		AuCl_3_	30.5 ± 2.2 nm	6.3 ± 2.4 nm

Pd mineralization occurred exclusively on TMV2Cys and BSMV when the biotemplates were incubated in Pd precursor solutions containing chlorides, including Na_2_PdCl_4_, K_2_PdCl_4_, and PdCl_2_. This phenomenon is attributed to the adsorption of metal precursor ions onto the CPs of the biotemplates through the ligand-switching mechanism of chlorides.^18^ Na_2_PdCl_4_, K_2_PdCl_4_, and PdCl_2_ dissociates into PdCl_4_^2–^ ions in aqueous solution. PdCl_4_^2–^ is a decahedral ion with four chlorides connected to a central Pd atom. The chlorides of PdCl_4_^2–^ participate in ligand exchange with the functional groups of the amino acid residues present on the CPs of TMV2Cys and BSMV; thereby, adsorbing the PdCl_4_^2–^ ions onto the outer surface of the virions. The adsorbed PdCl_4_^2–^ ions are converted into Pd atoms by the electron-rich functional groups such as amine and carboxyl groups present in amino acid residues; thereby, forming a Pd coating on the CPs of TMV2Cys and BSMV. Na_2_PdBr_4_ dissociates into PdBr_4_^2–^ ions that lack the chlorides necessary to adsorb themselves onto the outer surface of TMV2Cys and BSMV; therefore, preventing Pd mineralization.

BSMV-Pd virions were thicker than those of TMV2Cys-Pd. BSMV isoelectric point is 4.5 compared to that of TMV2Cys at 3.5; therefore, there are more positively charged functionalities in BSMV.^40,41^ The positively charged functionalities of BSMV permit the adsorption of metal precursor ions onto the CPs using both electrostatic and covalent interactions; therefore, resulting in the adsorption of more Pd precursor ions. TMV2Cys depends solely on covalent interactions; therefore, fewer Pd precursor ions are adsorbed during incubation.^42^ BSMV-Pd and TMV2Cys-Pd, produced by incubating BSMV and TMV2Cys in PdCl_2_, exhibit thicker layers of Pd mineralization compared to BSMV-Pd and TMV2Cys-Pd produced using Na_2_PdCl_4_ and K_2_PdCl_4_ solutions. Chloride ions are released into the aqueous solution after the PdCl_4_^2–^ ions are converted to Pd atoms by the amino acid residues of the CPs. As a result, a 0.75 mM PdCl_2_ solution has a lower concentration of chloride ions compared to 0.75 mM Na_2_PdCl_4_ and K_2_PdCl_4_ solutions.^43^ The chloride ions present in the incubation solution react with the amino acid residues of TMV2Cys and BSMV CPs, leading to the chlorination of these amino acid residues.^44^ The higher concentration of chloride ions in the Na_2_PdCl_4_ and K_2_PdCl_4_ solutions results in comparatively greater number of amino acid residues of TMV2Cys and BSMV being chlorinated during incubation. Chlorinated amino acid residues are unable to participate in the ligand-switching process necessary for PdCl_4_^2–^ ions to adsorb onto biotemplates.

### 2.3 Kinetic Study of Palladium Precursor Reduction on TMV2Cys and BSMV

**Figure 3:**
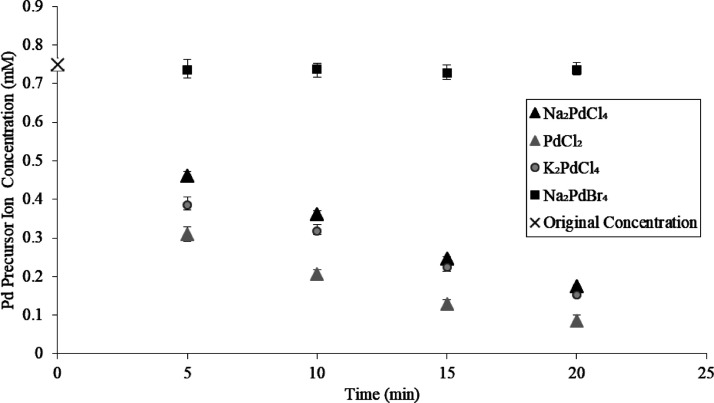
Pd precursor ion concentration versus time for BSMV incubated in Na_2_PdCl_4_, K_2_PdCl_4_, Na_2_PdBr_4_, and PdCl_2_ solution.

**Figure 4:**
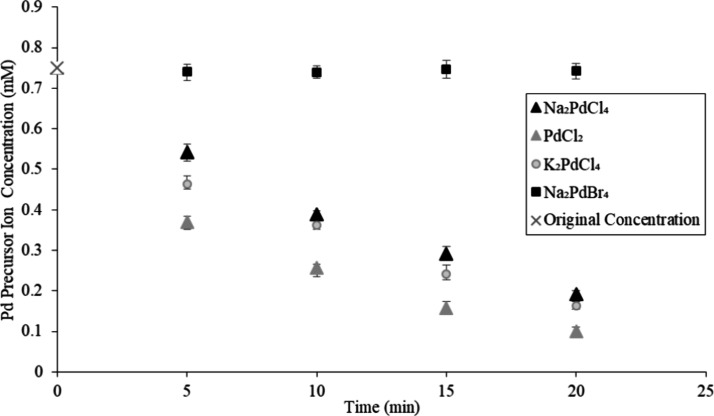
Pd precursor ion concentration versus time for TMV2Cys incubated in a Na_2_PdCl_4_, K_2_PdCl_4_, Na_2_PdBr_4_, and PdCl_2_ solution.

A kinetic study was conducted to examine the mechanism of adsorption of Pd precursor ions onto TMV2Cys and BSMV biotemplates. Pd mineralization reactions were conducted with a spectrophotometer at 55 °C. UV–vis readings were taken in 5 min intervals, and the intensity of the absorption peak at 425 nm (corresponding to the PdCl_4_^2–^ ion precursor) was converted to PdCl_4_^2–^ ion concentration using the calibration curve. The concentration of PdCl_4_^2–^ ions recorded at a specific time represents the Pd precursor remaining in the reaction solution that has not yet mineralized on the CPs of TMV2Cys or BSMV. The change in the concentration of PdCl_4_^2–^ ions with time demonstrates the rate at which PdCl_4_^2–^ ions are adsorbed onto the viral biotemplates. The concentration of Pd precursor ions versus time for BSMV incubated in Na_2_PdCl_4_, K_2_PdCl_4_, Na_2_PdBr_4_, and PdCl_2_ solutions is displayed in Figure 3, and the concentration of Pd precursor ions versus time for TMV2Cys incubated in Na_2_PdCl_4_, K_2_PdCl_4_, Na_2_PdBr_4_, and PdCl_2_ solution is displayed in Figure 4.

Based on Figures 3 and 4, PdCl_4_^2–^ ions were adsorbed rapidly by the BSMV and TMV2Cys during the first 5 min of the incubation, followed by a more gradual rate of PdCl_4_^2–^ ion adsorption during the remaining 15 min of incubation. The initially rapid rate of PdCl_4_^2–^ ion adsorption is due to the large number of amino acid residues available for metal mineralization on the CPs of BSMV and TMV2Cys that adsorb the PdCl_4_^2–^.^18^ As the incubation period progresses, Pd covers a greater number of sites on the CPs. Consequently, fewer amino acid residues remain available to adsorb PdCl_4_^2–^ ions, leading to a reduction in the rate of PdCl_4_^2–^ ion adsorption after the initial 5 min of incubation.

PdCl_4_^2–^ ions were adsorbed faster by BSMV compared to that by TMV2Cys. PdCl_4_^2–^ ion concentration after the first 5 min of incubation is about 20% lower when BSMV is incubated in Pd precursor solutions, compared to TMV2Cys. BSMV virions use both electrostatic and covalent interactions to adsorb PdCl_4_^2–^ ions onto the CPs of the virions, unlike TMV2Cys which depends solely on covalent interactions to adsorb PdCl_4_^2–^ ions; therefore, BSMV adsorbs precursor ions faster compared to TMV2Cys. PdCl_2_ solution contains a lower concentration of chloride ions compared to Na_2_PdCl_4_ and K_2_PdCl_4_ solutions; therefore, fewer amino acid residues are chlorinated when BSMV and TMV2Cys are incubated in PdCl_2_.

Based on Figures 3 and 4, the adsorption of PdCl_4_^2–^ ions occurred most rapidly when BSMV and TMV2Cys were incubated in a PdCl_2_ solution. This can be attributed to the higher availability of amino acid residues for adsorbing PdCl_4_^2–^ ions. According to Figures 3 and 4, the concentration of PdBr_4_^2–^ ions remained constant during the 20 min incubation period. The PdBr_4_^2–^ ions lack chlorides required for ligand switching necessary to adsorb the PdBr_4_^2–^ ions onto the outer surfaces of BSMV and TMC2Cys.

### 2.4 Platinum and Gold Mineralization on TMV2Cys and BSMV

**Figure 5:**
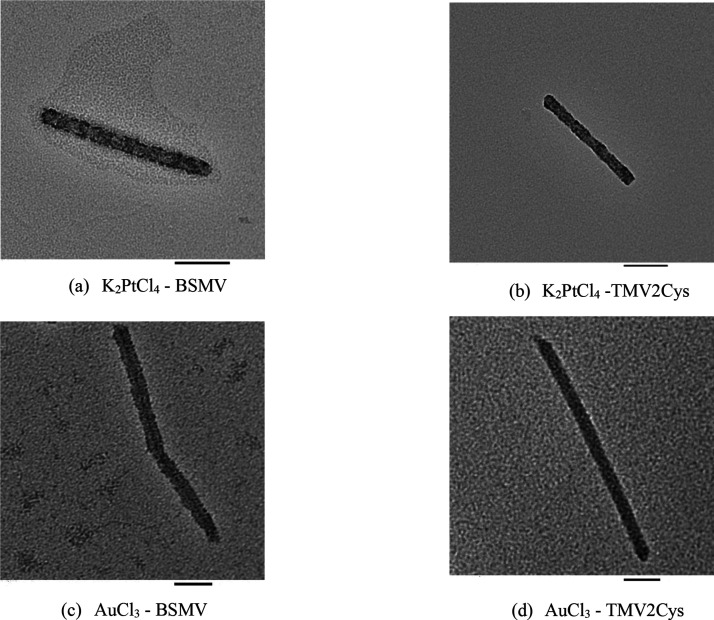
TEM images of BSMV-Pt produced by incubating BSMV in K_2_PtCl_4_ (a), TMV2Cys-Pt produced by incubating TMV2Cys in K_2_PtCl_4_ (b), BSMV-Au produced by incubating BSMV in AuCl_3_ (c), and TMV2Cys-Au produced by incubating TMV2Cys in AuCl_3_ (d) (scale bars: 100 nm).

TMV2Cys and BSMV biotemplates were incubated in solutions of K_2_PtCl_4_ and AuCl_3_ to mineralize Pt and Au on their CPs, respectively. The BSMV-Pt, BSMV-Au, TMV2Cys-Pt, and TMV2Cys-Au samples generated from the reactions were subjected to TEM microscopy for the acquisition of the images depicted in Figure 5. The thickness of Pt and Au mineralization on the biotemplates was measured and is provided in Table 1. Based on Table 1, the layers of Pt mineralization on BSMV-Pt and TMV2Cys-Pt were thicker compared to Pd mineralization on BSMV-Pd and TMV2Cys-Pd. The layers of Au mineralization on BSMV-Au and TMV2Cys-Au were thicker compared with Pt mineralization on BSMV-Pt and TMV2Cys-Pt. The metals that dissociate into ions with more positive reduction potentials are more easily mineralized on the CPs of the BSMV and TMV2Cys.^33^ Thicker layers of metal coating were achieved when BSMV or TMV2Cys was incubated in K_2_PtCl_4_, compared to BSMV or TMV2Cys incubated in Pd precursors, because Pt^2+^ ions have a more positive reduction potential than Pd^2+^ ions. Similarly, thicker layers of metal coating were achieved when biotemplates were incubated in AuCl_3_, compared to biotemplates incubated in K_2_PtCl_4_.

BSMV-Pt and BSMV-Au exhibited thicker metal coatings compared to those of TMV2Cys-Pt and TMV2Cys-Au. This difference is attributed to BSMV having a higher isoelectric point than TMV2Cys, resulting in a greater abundance of positively charged functionalities on BSMV.^40,41^ The positive functionalities of BSMV facilitate the adsorption of metal precursor ions onto the CPs through a combination of electrostatic and covalent interactions, leading to the increased adsorption of metal precursor ions. TMV2Cys relies exclusively on covalent interactions, resulting in fewer Pt and Au precursor ions being adsorbed during incubation. This led to a thinner Pt or Au coating compared to BSMV-Pt and BSMV-Au.^42^

### 2.5 Kinetic Study of Platinum and Gold Precursor Reduction on TMV2Cys and BSMV

**Figure 6:**
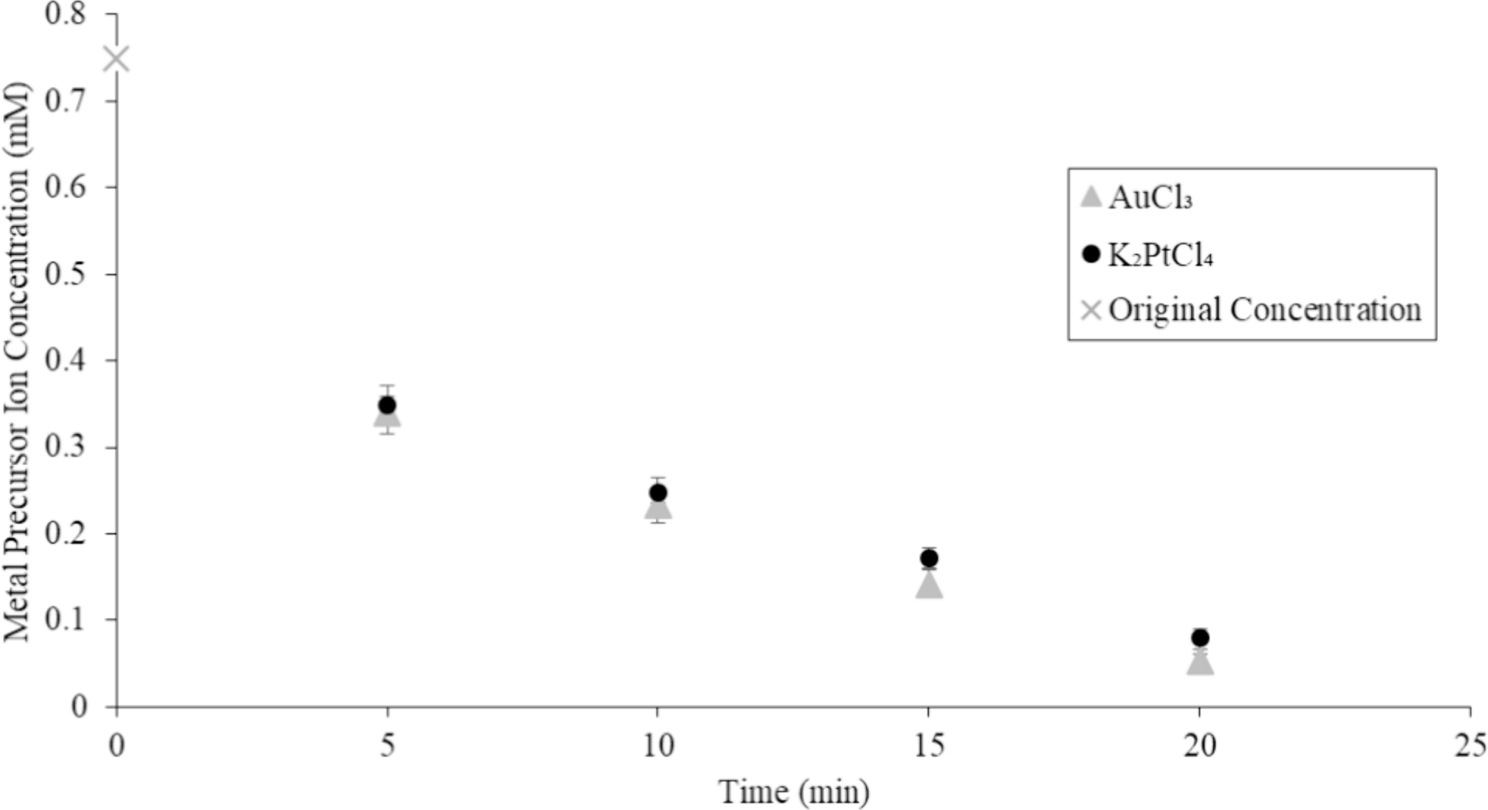
Metal precursor ion concentration versus time for BSMV incubated in a K_2_PtCl_4_ and AuCl_3_ solution.

**Figure 7:**
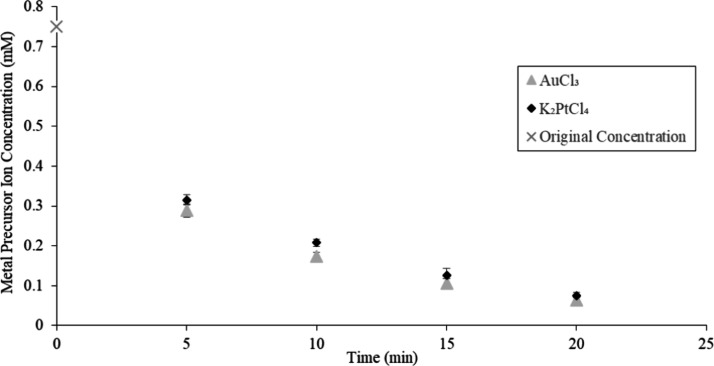
Metal precursor ion concentration versus time for TMV2Cys incubated in K_2_PtCl_4_ and AuCl_3_ solution.

A kinetic study was conducted to examine the adsorption mechanism of Pt and Au precursor ions by TMV2Cys and BSMV biotemplates. TMV2Cys and BSMV were incubated in K_2_PtCl_4_ and AuCl_3_ solutions at 55 °C in the reaction chamber of a UV–vis spectrophotometer. UV–vis readings were taken in 5 min intervals. The absorption peak at 388 nm observed in UV–vis spectra from the reactions using K_2_PtCl_4_ corresponds to the concentration of PtCl_4_^2–^ ions in the solution. The UV–vis absorption peak at 290 nm corresponds to the concentration of AuCl_4_^–^ ions in the solution for the reaction using AuCl_3_. The absorption values of peaks at 388 and 290 nm were converted to the PtCl_4_^2–^ and AuCl_4_^–^ concentrations, respectively, using the corresponding calibration curves. The concentration of PtCl_4_^2–^ and AuCl_4_^–^ ions recorded at a specific time represents the metal precursor remaining in the reaction solution that had not yet mineralized on the CPs of TMV2Cys or BSMV. The change in the concentration of PtCl_4_^2–^ and AuCl_4_^–^ ions with time demonstrates the rate at which these ions are adsorbed onto the viral biotemplates. The concentration of metal precursor ions versus time for BSMV incubated in K_2_PtCl_4_ and AuCl_3_ solutions is displayed in Figure 6. The concentration of metal precursor ions versus time for TMV2Cys incubated in K_2_PtCl_4_ and AuCl_3_ is illustrated in Figure 7.

Based on Figures 6 and 7, PtCl_4_^2–^ and AuCl_4_^–^ ions were adsorbed rapidly by BSMV and TMV2Cys during the first 5 min of incubation, followed by a more gradual rate of PtCl_4_^2–^ and AuCl_4_^–^ ions adsorption during the remaining 15 min of incubation. The initial rapid rates of PtCl_4_^2–^ and AuCl_4_^–^ can be attributed to the abundance of amino acid residues on the CPs of BSMV and TMV2Cys. These residues effectively adsorb the PtCl_4_^2–^ and AuCl_4_^–^ ions.^18^ More amino acid residues on the CPs are covered by Pt and Au as the incubation period progresses. When there are fewer sites available to adsorb the metal precursor ions, the rate of PtCl_4_^2–^ and AuCl_4_^–^ ions adsorption decreases after the initial 5 min of incubation. PtCl_4_^2–^ and AuCl_4_^–^ ions were adsorbed faster by BSMV compared to TMV2Cys. BSMV virions use both electrostatic and covalent interactions to adsorb PtCl_4_^2–^ and AuCl_4_^–^ ions onto the CPs of the virions, unlike TMV2Cys which depends solely on covalent interactions to adsorb PtCl_4_^2–^ and AuCl_4_^–^ ions. The AuCl_4_^–^ ions were adsorbed faster by BSMV and TMV2Cys compared to PtCl_4_^2–^ ions because Au mineralizes at a faster rate compared to Pt on the BSMV and TMV2Cys CPs. As the incubation period progresses, a higher rate of mineralization for gold (Au) compared with platinum (Pt) results in a more rapid decline in the concentration of AuCl_4_^–^ ions near the surface of the biotemplates. Consequently, this differential rate of mineralization causes the AuCl_4_^–^ ions to diffuse faster toward the biotemplates compared to PtCl_4_^2–^.^45^ The faster rate of ion diffusion contributes to the faster adsorption of AuCl_4_^–^ ions by BSMV and TMV2Cys, compared to the rate at which PtCl_4_^2–^ ions are adsorbed. Pt mineralization was thicker on BSMV-Pt and TMV2Cys-Pt, compared to Pd mineralization on BSMV-Pd and TMV2Cys-Pd. Pt mineralizes at a rate faster than that of Pd; therefore, PtCl_4_^2–^ diffuses faster than PdCl_4_^2–^. PtCl_4_^2–^ was adsorbed by BSMV and TMV2Cys at a faster rate compared to PdCl_4_^2–^ due to the faster mineralization of Pt compared to that of Pd. The faster rate of PtCl_4_^2–^ adsorption by biotemplates resulted in thicker layers of Pt on BSMV-Pt and TMV2Cys-Pt, compared to Pd mineralization on BSMV-Pd and TMV2Cys-Pd.

### 2.6 Thermal Annealing of Pd-Coated TMV2Cys

**Figure 8:**
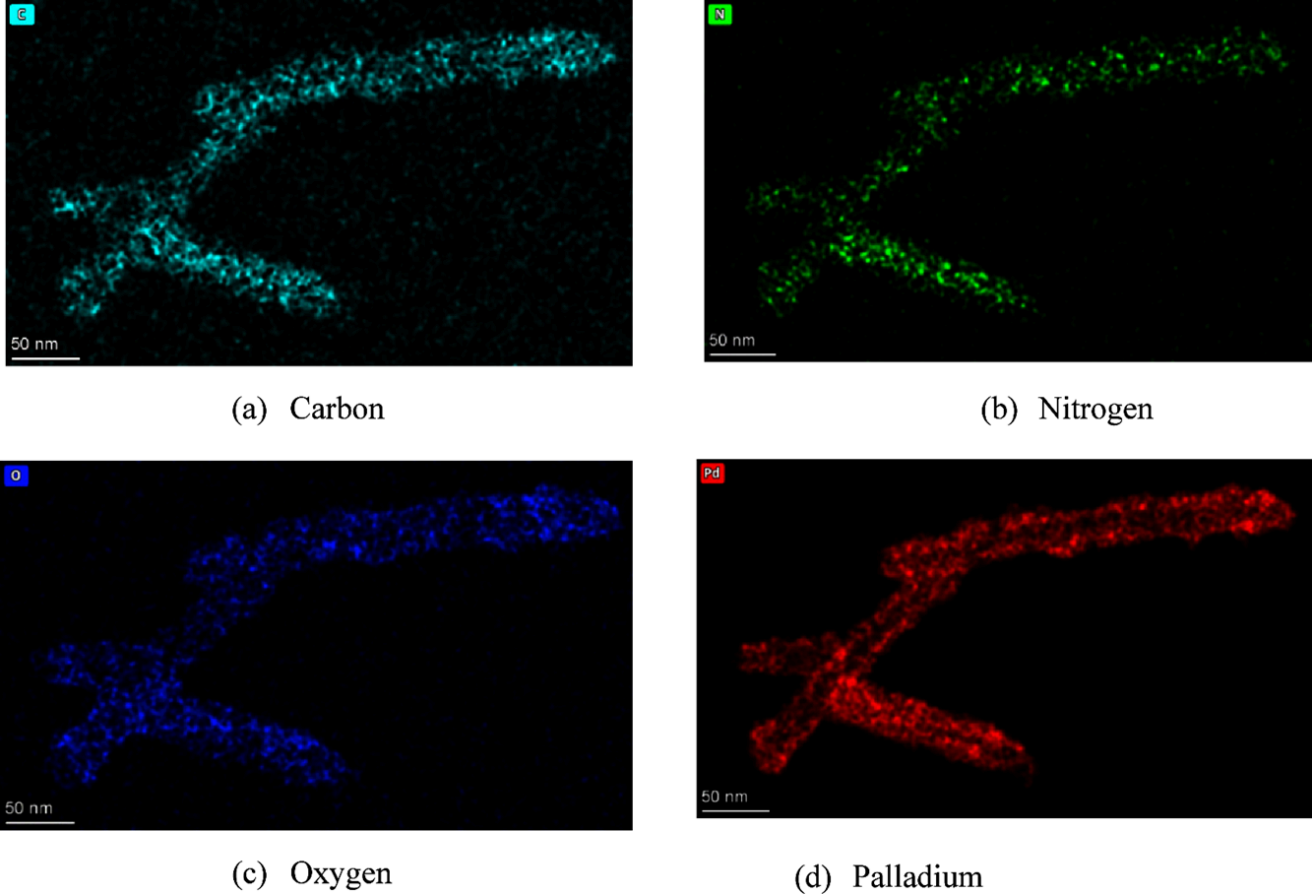
EDS mapping of TMV2Cys-Pd coated with five cycles of Pd coating.

To achieve a thicker layer of metal mineralization suitable for thermal annealing, five cycles of Pd mineralization was conducted. The resulting TMV2Cys-Pd was characterized by using EDS prior to thermal annealing, as illustrated in Figure 8. EDS mapping was also conducted to investigate the elemental composition of the Pd-coated TMV, as displayed in Figure 8. The presence of carbon, oxygen, and nitrogen, confirmed by Figures 8a, b, and c, respectively, signifies that the biotemplate is composed of proteins. The presence of the mineralized Pd coating is also confirmed, as shown in Figure 8d.

**Figure 9:**
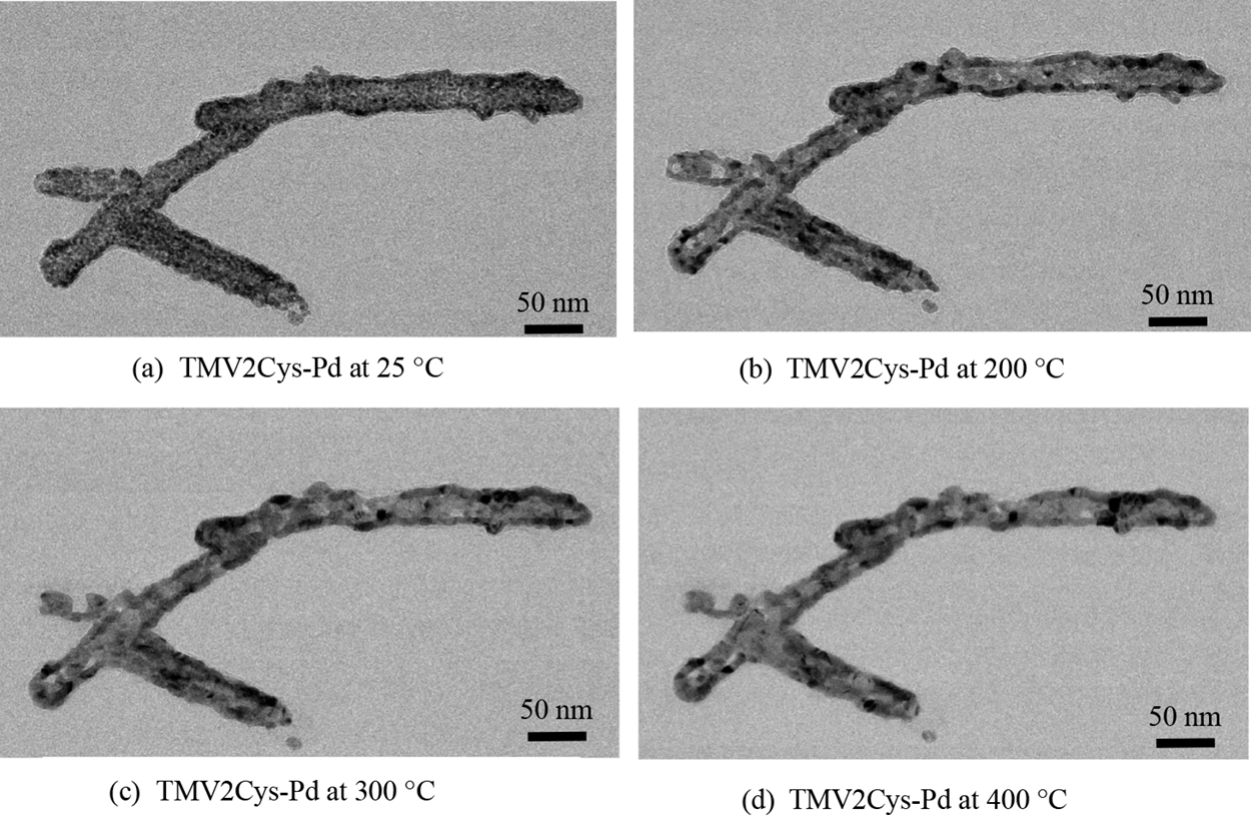
TEM images of TMV2Cys-Pd with five layers of coating at different temperatures during *in situ* TEM.

Surface changes on the virion were observed at approximately 200 °C, where smaller particles began to coalesce into larger ones. The particle sizes continued to increase as the temperature was raised to 300 and 400 °C, as depicted in Figure 9. This phenomenon can be attributed to Ostwald ripening, wherein larger particles grow at the expense of smaller ones in pursuit of a more thermodynamically favorable state. The resulting larger particles reduce the number of grain boundaries present on the Pd coating, thereby improving conductivity.

The surface of the TMV2Cys with Pd coating appears smoothest at 200 °C, despite the formation of fewer grain boundaries at 300 and 400 °C due to the aggregation of larger particles. The smooth surface observed at 200 °C makes it the appropriate temperature for annealing Pd-coated TMV2Cys.

## 3 Conclusions

Based on the findings of this study, noble metal mineralization was observed when TMV2Cys and BSMV were incubated in aqueous solutions of Na_2_PdCl_4_, K_2_PdCl_4_, PdCl_2_, K_2_PtCl_4_, and AuCl_3_. Specifically, BSMV-Pd and TMV2Cys-Pd were produced when BSMV and TMV2Cys were incubated in Pd precursors, such as Na_2_PdCl_4_, K_2_PdCl_4_, and PdCl_2_. However, when BSMV and TMV2Cys were incubated in a Na_2_PdBr_4_ solution, Pd mineralization did not occur. This is because Na_2_PdBr_4_ dissociates into PdBr_4_^2–^ ions, which contain bromide ligands. Metal ion complexes with bromide ligands have reduction potentials lower than those containing chlorides; therefore, amino acid residues are unable to reduce PdBr_4_^2–^ ions to cause Pd mineralization. TMV2Cys and BSMV effectively adsorb only salts containing chlorides. Therefore, Na_2_PdCl_4_, K_2_PdCl_4_, and PdCl_2_ are suitable candidates for depositing Pd on TMV2Cys and BSMV, as these salts dissociate into PdCl_4_^2–^ ions. The thickest coating of Pd mineralization was produced when TMV2Cys or BSMV were incubated in PdCl_2_ because fewer chloride ions were present in its solution compared to Na_2_PdCl_4_ and K_2_PdCl_4_. The lower occurrence of chloride ions reduces the chlorination of amino acid residues present on TMV2Cys and BSMV CPs; therefore, more amino acid residues are available to adsorb PdCl_4_^2–^ ions when TMV2Cys and BSMV are incubated in PdCl_2_. Thicker Pd coating was observed when BSMV was incubated in Pd precursor solutions because BSMV uses both electrostatic and covalent interactions to adsorb the precursor ions onto its CPs, unlike TMV which depends solely on covalent interactions. K_2_PtCl_4_ and AuCl_3_ were chosen as the precursor salts for mineralizing Pt and Au on TMV2Cys and BSMV CPs. K_2_PtCl_4_ and AuCl_3_ are soluble in aqueous solution and dissociate into PtCl_4_^2–^ and AuCl_4_^–^ ions, respectively. Both PtCl_4_^2–^ and AuCl_4_^–^ ions contain chlorides that participate in the ligand-switching process, which cause the ions to adsorb onto TMV2Cys and BSMV during incubation. BSMV-Pt and TMV2Cys-Pt were successfully produced by incubating BSMV and TMV2Cys in K_2_PtCl_4_. BSMV-Au and TMV2Cys-Au were successfully produced by incubating BSMV and TMV2Cys in AuCl_3_. There were thicker layers of Au mineralization on BSMV-Au and TMV2Cys-Au, compared to Pt mineralization on BSMV-Pt and TMV2Cys-Pt. BSMV-Pt and TMV2Cys-Pt had thicker layers of Pt mineralization compared to those of BSMV-Pd and TMV2Cys-Pd. Au mineralizing on BSMV or TMV2Cys CPs at a faster rate compared with Pt resulted in faster adsorption of Au relative to Pt. The more rapid adsorption rates produced thicker layers of metal mineralization. TMV2Cys-Pd was also annealed and characterized using *in situ* heating TEM, and it was demonstrated that the PdNRs with best surface smoothness were achieved at a temperature of around 200 °C.

Future research on metal NR synthesis using TMV2Cys and BSMV should explore their applications in various novel areas. This includes their utilization as nanocatalysts with the organic core serving as the substrate for supporting noble metal particles for catalytic applications. Additionally, conducting X-ray photoelectron spectroscopy, X-ray diffraction, and X-ray absorption spectroscopy will further elucidate the structural and oxidation state information on the Pd coatings, while providing deeper insights into the mineralization mechanism. Optimization of thermal annealing processes can be pursued to convert the metal-coated biotemplates into single-crystal metal NRs; thereby producing noble metal–carbon electrodes suitable for electronic applications. One potential application is the formulation of conductive inks and the manufacturing of transparent conductive patterns using thermally annealed noble metal-coated TMV and BSMV. Additionally, parametric studies are essential for the mineralization of Au and Pt on TMV2Cys and BSMV, as there is limited scientific literature on coating viral biotemplates with these noble metals compared to the more commonly studied Pd. VLPs of TMV2Cys and BSMV can be produced in bacteria, such as *Escherichia coli* (*E. coli*), with designer CPs, which could not be produced in plants because they would be noninfectious. The combination of VLPs with the methods established in this study, along with biotemplate removal through thermal annealing, represents the comprehensive series of steps required for the sustainable production of noble metal NRs using rod-shape viral biotemplates.

## 4 Experimental Details

### 4.1 Materials and Reagents

Material and reagents included sodium tetrachloropalladate (Na_2_PdCl_4_) (Sigma-Aldrich, 1003067874), potassium tetrachloropalladate (K_2_PdCl_4_) (Sigma-Aldrich, 1003332735), sodium tetrabromopalladate (Na_2_PdBr_4_) (Alfa Aesar, 50495131), palladium(II) chloride (PdCl_2_) (Sigma-Aldrich, 1003359585), potassium tetrachloroplatinate (K_2_PtCl_4_) (Sigma-Aldrich 1003503000), gold(III) chloride (AuCl_3_) (Sigma-Aldrich, 1003588143), sodium borate (CAS Number: 1303-96-4), Triton X-100 (CAS Number: 9036-19-5), β-mercaptoethanol (CAS Number: 60-24-2), chloroform (CAS Number: 67-66-3), poly(ethylene glycol) (PEG-8000) (CAS Number: 25322-68-3), disodium phosphate (Na_2_HPO_4_, CAS Number: 7558-79-4), sodium ascorbate (CAS Number: 134-03-2), Celite 545 (CAS Number: 68855-54-9), sodium chloride (NaCl) (CAS Number: 7647-14-5), tris(hydroxymethyl)aminomethane (tris) (CAS Number: 77-86-1), ethylenediaminetetraacetic acid (EDTA) (CAS Number: 60-00-4), and deionized water (DIW) (LabChem, LC267505). Na_2_PdCl_4_, K_2_PdCl_4_, Na_2_PdBr_4_, and PdCl_2_ were the precursor salts used for preparing the solutions in which viral biotemplates were incubated for Pd mineralization. PtCl_2_ and AuCl_3_ served as the precursor salts for preparing the solutions in which viral biotemplates were incubated for Pt and Au mineralization, respectively. Each metal precursor was dissolved in DIW to create 0.75 mM solutions of Na_2_PdCl_4_, K_2_PdCl_4_, Na_2_PdBr_4_, PdCl_2_, K_2_PtCl_4_, and AuCl_3._

### 4.2 Production of TMV2Cys and BSMV Biotemplates

Tobacco plants were inoculated with TMV2Cys and barley plants were inoculated with BSMV. Both viruses were isolated according to published methods and suspended in Tris-HCl buffer.^37−39^

### 4.3 Depositing Palladium, Platinum, and Gold on TMV2Cys and BSMV

**Figure 10:**
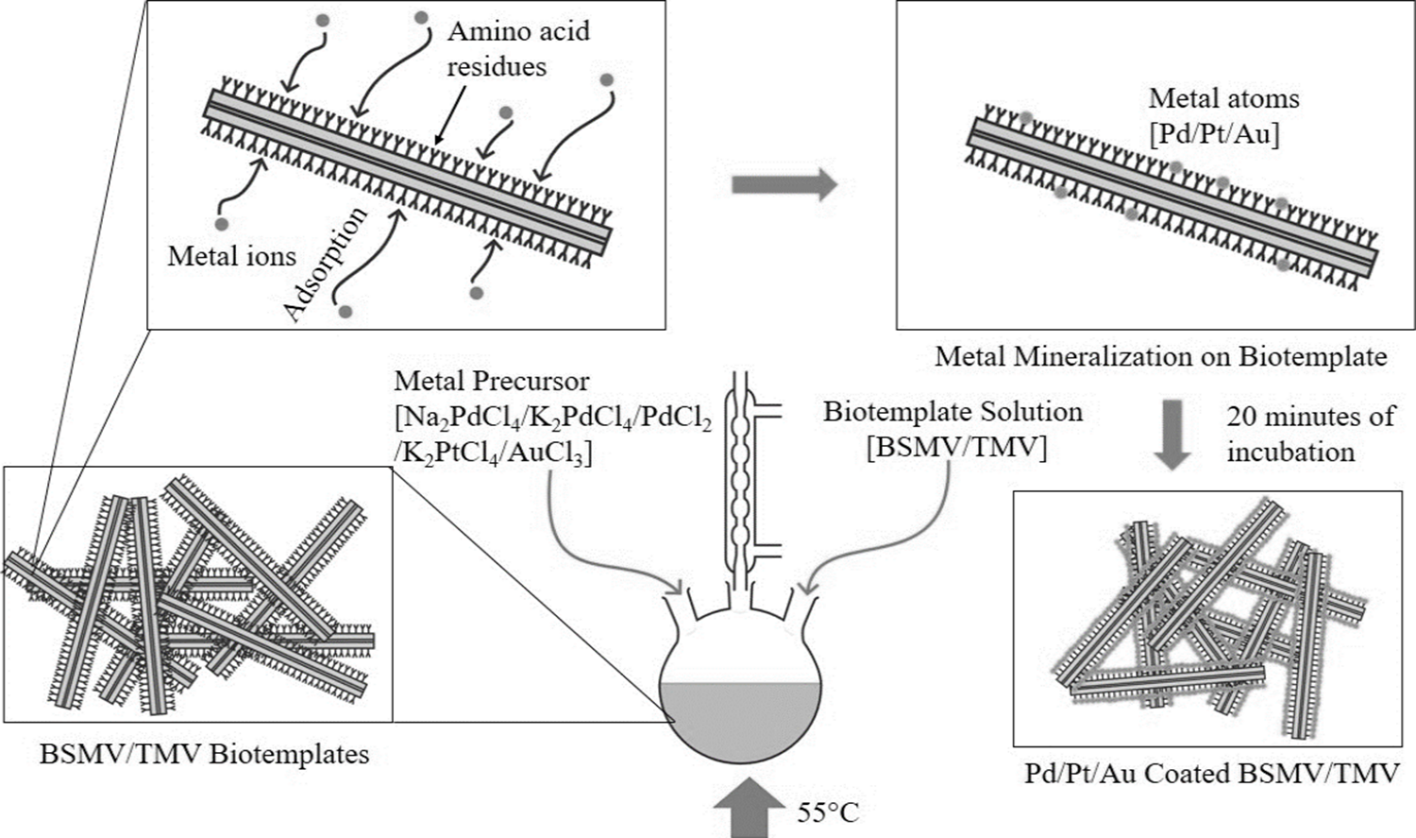
Schematic of depositing Pd, Pt, and Au on TMV2Cys and BSMV via incubation in the corresponding metal precursor solutions.

1 mL of a 0.035 mg/mL solution of TMV2Cys or BSMV was heated in a three-neck flask placed in a water bath at a temperature of 55 °C, as schematically illustrated in Figure 10. 0.75 mL of a 0.75 mM metal precursor solution consisting of either Na_2_PdCl_4_, K_2_PdCl_4_, Na_2_PdBr_4_, PdCl_2_, PtCl_2_, or AuCl_3_ was pipetted into the reaction vessel containing TMV2Cys or BSMV after it had been heated for 2 min at 55 °C. The metal precursor and biotemplate solutions were held for 20 min at 55 °C, and the reaction was quenched by placing the flask in an ice bath. No Pd mineralization occurred when TMV2Cys and BSMV were incubated in a Na_2_PdBr_4_ solution. To prepare samples for thermal annealing using *in situ* heating TEM, five cycles of Pd coating on TMV2Cys were conducted by washing the virions obtained from the previous cycle with DIW. Subsequently, the virions were introduced in a 0.75 mM solution of PdCl_2_ each time for 20 min to prepare the Pd-TMV2Cys.

### 4.4 Final Sample Washing Procedure

The quenched reaction solution was transferred to a 25 mL plastic vial, followed by the addition of 10 mL of DIW. The vial was then gently swirled for 2 min. Virions with metal mineralization were subsequently allowed to settle down at the bottom of the vial. Water wash solution (10 mL) was carefully pipetted from the top of the sample, leaving the virions undisturbed at the bottom of the vial. The washing process was repeated four times for each sample obtained from the reactions, and the resulting virions were characterized using TEM. The TMV2Cys and BSMV that were incubated in Na_2_PdBr_4_ did not settle at the bottom of the vial due to lack of Pd mineralization on their CPs; therefore, the viruses incubated in Na_2_PdBr_4_ could not be collected for TEM characterization.

### 4.5 Kinetic Study

Kinetic studies were conducted by incubating TMV2Cys and BSMV with metal precursor solutions in a glass cuvette placed in the temperature-controlled reactor chamber of the UV–vis spectrophotometer at 55 °C. 1 mL of 0.035 mg/mL solution of TMV2Cys or BSMV solution was warmed in the glass cuvette for two min at 55 °C. Then, 0.75 mL of a 0.75 mM metal precursor solution consisting of either Na_2_PdCl_4_, K_2_PdCl_4_, Na_2_PdBr_4_, PdCl_2_, K_2_PtCl_4_, or AuCl_3_ was pipetted into the cuvette containing MV2Cys or BSMV. The reaction solution was held for 20 min at 55 °C, and UV–vis spectroscopic readings were obtained at 5 min intervals. Na_2_PdCl_4_, K_2_PdCl_4_, and PdCl_2_ dissociate into PdCl_4_^2–^ ions, Na_2_PdBr_4_ dissociates into PdBr_4_^2–^ ions, K_2_PtCl_4_ dissociates into PtCl_4_^2–^ ions in aqueous solution, and AuCl_3_ dissociates into AuCl_4_^–^ ions in aqueous solution containing Tris-HCl buffer. PdCl_4_^2–^, PdBr_4_^2–^, PtCl_4_^2–^, and AuCl_4_^–^ ions have characteristic UV–vis absorbance peaks at 425, 332, 388, and 290 nm, respectively. UV–vis absorbance values were converted into PdCl_4_^2–^, PdBr_4_^2–^, PtCl_4_^2–^, and AuCl_4_^–^ concentration values by using a calibration curve for each salt precursor. UV–vis absorbance of Na_2_PdCl_4_, K_2_PdCl_4_, Na_2_PdBr_4_, PdCl_2_, K_2_PtCl_4_, and AuCl_3_ solutions with 0.25, 0.50, 1, and 2 mM concentrations were used to create the calibration curves. The results were used to create graphs of precursor ion concentration versus time.

### 4.6 Characterization

TEM imaging was performed by a Jeol JEM 2100 microscope on carbon-coated copper grids at 200 kV. A washed sample (5 μL) was pipetted onto the grid and allowed to dry at room temperature. The samples were subsequently negatively stained using uranyl acetate and loaded onto a TEM microscope for characterization. The diameters of TMV2Cys and BSMV with metal mineralization were measured using the Image-J software. To obtain an average diameter, the diameter of biotemplates with metal mineralization was measured for all virions in three different TEM images. About 15 to 20 virions were measured from each type of TMV2Cys and BSMV with metal mineralization to find their average diameters. UV–vis characterization for the kinetic study was conducted by using a Mettler Toledo UV 5 UV–vis spectrophotometer. EDS characterization was conducted by using a JEM 2100 microscope. EDS mapping was generated using the F200X TEM instrument, both before annealing and after the annealed samples were cooled to room temperature.

### 4.7 Thermal Annealing

Thermal annealing of TMV2Cys coated with five cycles of Pd was conducted using *in situ* TEM, in an FEI Talos F200X TEM instrument. A washed sample (5 μL) was pipetted onto a ceramic specimen holder and allowed to dry at room temperature. Subsequently, the specimen holder containing the sample was mounted onto a Protochips Heating TEM Fusion holder and then loaded onto the TEM microscope stage. The Pd-coated TMV2Cys was located, and the temperature was gradually increased to the desired level for thermal annealing at a ramp rate of 1 °C/s.
